# P-WIN deployment: real-time personalized machine learning to predict critical deterioration in a pediatric intensive care unit

**DOI:** 10.1093/jamiaopen/ooag202

**Published:** 2026-10-19

**Authors:** Eamonn Tweedy, Sanjiv Mehta, Sachin Grover, Hannah R Stinson, Victor M Ruiz, Erika Pleskunas, James Sannino, Akira Nishisaki, Fuchiang (Rich) Tsui

**Affiliations:** Tsui Laboratory, Department of Biomedical and Health Informatics, Children’s Hospital of Philadelphia, Philadelphia, PA 19146, United States; Division of Critical Care Medicine, Department of Anesthesiology and Critical Care Medicine, Children’s Hospital of Philadelphia, Philadelphia, PA 19104, United States; Department of Anesthesiology and Critical Care, University of Pennsylvania, Philadelphia, PA 19104, United States; Department of Biomedical and Health Informatics, Children’s Hospital of Philadelphia, Philadelphia, PA 19146, United States; Tsui Laboratory, Department of Biomedical and Health Informatics, Children’s Hospital of Philadelphia, Philadelphia, PA 19146, United States; Division of Critical Care Medicine, Department of Anesthesiology and Critical Care Medicine, Children’s Hospital of Philadelphia, Philadelphia, PA 19104, United States; Department of Anesthesiology and Critical Care, University of Pennsylvania, Philadelphia, PA 19104, United States; Tsui Laboratory, Department of Biomedical and Health Informatics, Children’s Hospital of Philadelphia, Philadelphia, PA 19146, United States; Digital and Technology Services, Children’s Hospital of Philadelphia, Philadelphia, PA 19104, United States; Center for Healthcare Quality and Analytics, Children’s Hospital of Philadelphia, Philadelphia, PA 19104, United States; Division of Critical Care Medicine, Department of Anesthesiology and Critical Care Medicine, Children’s Hospital of Philadelphia, Philadelphia, PA 19104, United States; Department of Anesthesiology and Critical Care, University of Pennsylvania, Philadelphia, PA 19104, United States; Tsui Laboratory, Department of Biomedical and Health Informatics, Children’s Hospital of Philadelphia, Philadelphia, PA 19146, United States; Division of Critical Care Medicine, Department of Anesthesiology and Critical Care Medicine, Children’s Hospital of Philadelphia, Philadelphia, PA 19104, United States; Department of Anesthesiology and Critical Care, University of Pennsylvania, Philadelphia, PA 19104, United States; Department of Biostatistics, Epidemiology, and Informatics, University of Pennsylvania, Philadelphia, PA 19104, United States

**Keywords:** critical care, pediatrics, deterioration, early warning system, machine learning

## Abstract

**Objectives:**

Early recognition of critical deterioration events (CDEs) in pediatric intensive care units (PICUs) is crucial to improving outcomes, but the real-time performance of machine-learning (ML)-based alert systems is rarely assessed. We prospectively evaluated the performance, operational utility, and deployment feasibility of the PICU Warning Index (P-WIN), a personalized ML alert system predicting CDE in the PICU.

**Materials and methods:**

In this prospective cohort study at a quaternary-care children’s hospital, P-WIN estimated deterioration risk for PICU patients aged 0-24 years at 30-minute intervals between January 7 and April 7, 2026 using electronic health record data collected via Fast Healthcare Interoperability Resources and proprietary EPIC API endpoints. CDEs included cardiopulmonary resuscitation, initiation of extracorporeal membrane oxygenation, dilute (10 μg/mL) epinephrine bolus, or unplanned endotracheal intubation. P-WIN was compared to baseline workflows: an automated PICU Warning Tool and a clinician-huddle-based assessment. Primary measures were CDE-level sensitivity, admission-level positive predictive value (PPV), and alerts per patient-day.

**Results:**

Among 1005 PICU patients, 99 CDEs were experienced by 62 patients. P-WIN detected 73% of these CDE 1-24 hours in advance, outperforming the PICU Warning Tool (39%, *P* < .05) and huddle workflow (23%, *P* < .05). P-WIN generated 0.21 (95% CI, 0.18-0.23) alerts per patient-day. The PICU Warning Tool and huddle workflow had similar PPV to P-WIN.

**Conclusion:**

P-WIN detected significantly more CDEs than existing PICU Warning tool and huddle workflows while maintaining a similar PPV. Our findings underscore the feasibility of ML-based personalized warning systems to enhance shared situational awareness of PICU deterioration.

## Introduction

Early identification of cardiopulmonary deterioration in the pediatric intensive care unit (PICU) remains a central challenge for critical care teams.^1^ Earlier recognition of high-risk patients may improve situational awareness among the clinical team and provide additional time for escalation of care and implementation of mitigation strategies. Prior situational awareness initiatives have been associated with reductions in unrecognized clinical deterioration, serious safety events, and patient harm.^2–6^ There is a growing body of work demonstrating that deterioration can often be identified hours before overt decompensation, creating opportunities for earlier intervention and improved outcomes.^7–10^

Prior systems have been limited by rule-based designs, insufficient temporal precision, or evaluation frameworks that incompletely characterize alert burden. Studies assessing the PICU Warning Tool, pCART, and pCREST systems reported operational metrics such as number needed to screen (NNS), number needed to alert (NNA), and positive predictive value (PPV), but these analyses were performed at the patient or admission level.^5^^,^^7^^,^^8^^,^^11^ Because clinicians interact with these systems on the level of individual alerts, alert volume estimates such as alert-level PPV or average number of alerts per patient-day may provide additional insight into alert burden and clinical utility which are essential factors for clinical adoption and sustainability.

Machine-learning (ML)-based prediction systems have shown improved performance compared to conventional models,^12^ but their prospective implementation and evaluation after model publication remain uncommon.^13^ Parts of the challenge stem from the technical integration and management of machine-learning inference with live electronic health record (EHR) systems and clinical usability of prediction systems.

High-dimensional ML models can provide increased performance when compared to smaller ML models, as well as the potential to identify highly personalized key risk drivers via SHapley Additive exPlanations (SHAP)^14^ analysis. However, deployment tends toward smaller models as relying on a large number of EHR variables could potentially exacerbate the technical challenges in real-world deployment.^15^^,^^16^

To address this gap, we silently deployed and evaluated the reviously validated PICU Warning INdex (P-WIN),^12^ a multi-prediction-horizon ensemble ML model predicting critical deterioration events (CDEs), operating in real time at a quaternary-care children’s hospital for 90 days, without delivering alerts to the EHR or the clinical team. We aimed to (1) prospectively evaluate P-WIN’s ability to detect CDE, (2) compare its performance and operational characteristics against 2 established PICU alert workflows, and (3) demonstrate the feasibility of deploying a personalized high-dimensional ML model in a real-time clinical setting.

## Methods

The Institutional Review Board at the Children’s Hospital of Philadelphia (CHOP) approved the study as exempt and waived informed consent (IRB 19017102). This study follows the Transparent Reporting of a Multivariable Prediction Model for Individual Prognosis or Diagnosis plus Artificial Intelligence (TRIPOD+AI).^17^

### Setting and population

The prospective cohort included all patients aged 0-24 years admitted to the CHOP Philadelphia Campus PICU department between January 7 and April 7, 2026. Some young adults are treated in the CHOP PICU, and we restricted to the same age range used in developing the P-WIN model.^12^ CHOP is a free-standing quaternary children’s hospital with approximately 100 licensed PICU beds, including a 25-bed section for chronically intubated or technology-dependent patients referred to as the progressive care unit (PCU). Consecutive PICU stays in which the patient left the PICU and returned within 24 hours were merged into continuous PICU admissions. The inclusion and exclusion process is illustrated in Figure 1.

**Figure 1.**
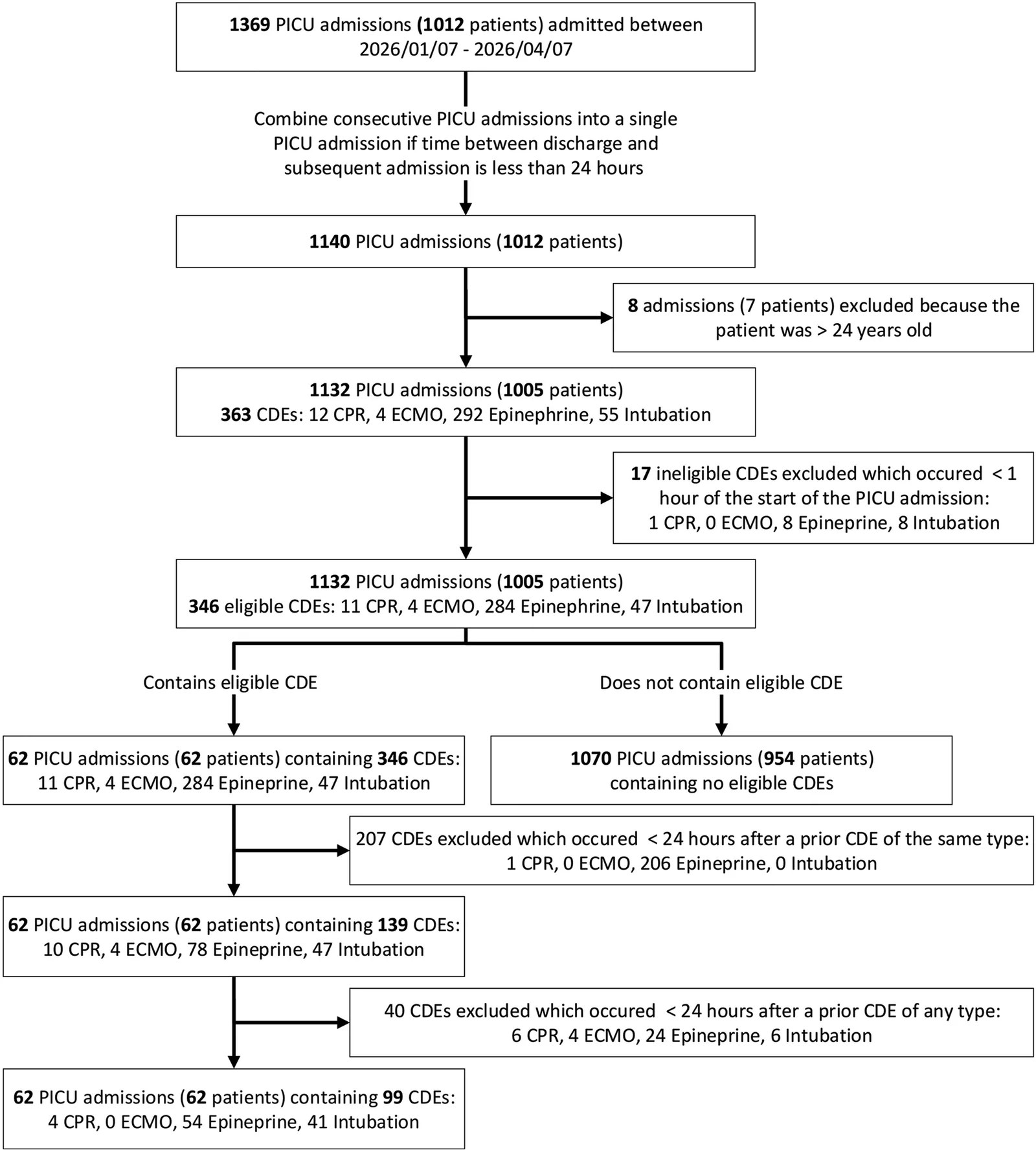
The inclusion and exclusion flow chart for the P-WIN real-time deployment study. CDE, critical deterioration event; CPR, cardiopulmonary resuscitation; ECMO, extracorporeal membrane oxygenation; intubation, unplanned endotracheal intubation.

### The P-WIN model and CDE outcome

The primary outcome was a composite of 4 CDE types: (1) cardiac arrest requiring cardiopulmonary resuscitation (CPR), (2) extracorporeal membrane oxygenation (ECMO) cannulation, (3) “dilute” (10 μg/mL concentration) epinephrine bolus, and (4) unplanned endotracheal intubation (henceforth called intubation), defined as any intubation that persisted for at least 48 hours to exclude elective/procedural airway management. CDEs were obtained from a local cardiac arrest database, local Virtual Pediatric Systems registry, and medication administration records. We consider a CDE to be detected by an alert system if there is an alert during the 1-24 hour window prior to the CDE, where the lower 1-hour limit was chosen as a minimal clinically actionable lead time and the 24-hour limit was selected to be consistent with published studies^11^^,^^12^ and accommodate comparators without defined horizons.

P-WIN is a validated ensemble of 6 XGBoost models predicting the risk of CDE 1, 2, 4, 6, 8, and 12 hours in advance.^12^ It uses 550 structured EHR variables (335 medication, 110 lab, 103 flowsheet, and 2 demographic variables). During the 2021-2022 retrospective assessment P-WIN detected 80% of CDEs, achieved an admission-level PPV of 0.11 (95% CI, 0.11-0.11), and generated 0.20 (95% CI, 0.19-0.21) alerts per patient-day.^12^ Admission-level PPV is defined to be the number of PICU admissions in which the first CDE was detected, divided by the number of PICU admissions having alerts. In the retrospective assessment^12^, 4% of admissions contained at least 1 CDE. P-WIN was intentionally trained using prediction horizons up to 12 hours, matching clinical actions within a shift and preserving strong discrimination at shorter horizons.

### Current state of the art workflows as comparators

We benchmarked P-WIN against the current PICU Warning Tool,^5^^,^^11^ an automated rules-based clinical decision support tool. In a 1-year 2-site evaluation, the PICU Warning Tool achieved an event-level sensitivity of 0.40 (95% CI, 0.33-0.49) and patient-level PPV of 0.07 (95% CI, 0.06-0.09).^11^

We also compared P-WIN performance to the best available proxy for human performance. Every 12 hours (8 AM and 8 PM), during unit-wide huddles, clinical teams identify patients who are at high risk of deterioration during the upcoming shift. This definition is more expansive than the P-WIN outcome (eg, can include high-risk extubations).

We also compared P-WIN’s performance during this prospective deployment to prior work,^12^ in which we assessed its performance and operational characteristics retrospectively using PICU admissions during 2021-2022.

### Real-time EHR data retrieval using APIs

EHR data were extracted every 30 minutes via a combination of Fast Healthcare Interoperability Resources (FHIR)^18^ and proprietary EPIC API pipelines,^19^ and PICU admissions were validated retrospectively using Admission, Discharge, and Transfer data. More details about API endpoints can be found in Supplementary Methods, and Figure 1 provides a visual summary of the data retrieval process. In cases of API request failure, we used cached data from the most recent timestamp, if available.

P-WIN model variables were extracted from medication, lab, flowsheet, and demographic data using the same procedure as during P-WIN model development.^12^ More details can be found in Supplementary Methods.

### Prediction frequency and alert policy

P-WIN generated predictions for cohort patients every 30 minutes. Silent alerts were generated at a pre-specified threshold which achieved 80% sensitivity during retrospective validation^12^ and were not displayed to clinicians. To minimize alert fatigue, we instituted a 12-hour refractory period following each alert. We excluded CDEs occurring in the first hour of a PICU admission as it’s not possible for an alert to detect these 1-24 hours in advance, and we excluded/censored subsequent CDEs within 24 hours of an initial CDE to avoid redundantly overcounting CDEs within the same deterioration trajectory. In contrast, when measuring sensitivity within individual CDE types, we only applied the 24-hour censoring among CDEs of the same type. The former censoring method resulted in the set of CDEs shown in the final box on the left side of Figure 1, whereas the latter censoring method resulted in the set in the penultimate box. As a sensitivity analysis, we also investigate thresholds that achieved 90% and 95% sensitivity in the 2021-2022 retrospective evaluation.^12^

### Evaluation metrics

We included 4 evaluation metrics for the prospective model deployment, namely (1) CDE-level sensitivity, (2) admission-level PPV, (3) alert-level PPV, and (4) average alert burden. Defining a true alert as an alert for which the patient has a CDE 1-24 hours following the alert, the admission-level PPV was the number of admissions where the first observed CDE had a true alert divided by the number of admissions having alerts. Alert-level PPV was the number of true alerts divided by the number of alerts. Finally, average alert burden was the average across all PICU admissions of the number of alerts divided by length of the admission in days. Although P-WIN is an ensemble of horizon models predicting CDEs up to 12 hours in advance, we adopted the 24-hour outcome window to maintain consistency with other deterioration risk studies.^11^ We assessed model calibration using the Brier Skill Score and a calibration plot. For the calibration assessment, we selected the prediction time closest to 2 hours prior to the first CDE for each admission with CDE(s), and a random prediction time for other admissions.

### Statistical analyses of model performance and real-time operations

We used 2000 bootstrap replicates to estimate 95% CIs, McNemar’s test^20^ for sensitivity differences, permutation tests^21^ with 10 000 iterates for PPV differences, and Wilcoxon’s signed rank test^22^ for alert burden and timeliness differences. We assessed whether the P-WIN model exhibited bias with respect to sex, race, ethnicity, or payor category by testing for differences in event-level sensitivity between groups using Fisher’s exact test.^23^

### Model explanations

To enhance clinical usability of P-WIN, we utilized SHapley Additive exPlanations (SHAP)^14^ values to provide real-time, patient-level feature attribution for each prediction.

### Sensitivity analysis of parsimonious P-WIN models

In this sensitivity analysis, we evaluated 3 parsimonious versions of the P-WIN model to test whether smaller models can approximate P-WIN’s real-time performance. Parsimonious models were developed using a similar methodology as the full P-WIN model but employing a top-*k* feature selector (*k* = 10, 20, 30) at each of the 6 prediction horizons. The resulting ensemble models rely on 12, 24, and 42 variables, respectively. The feature selector ranked variables by mutual information with the CDE outcome.

The parsimonious models all demonstrated statistically significantly lower AUROC performance than the full P-WIN model (*P* < .05) in our prior retrospective study.^12^

### Sensitivity analysis of excluding PCU admissions

As an additional sensitivity analysis, we recomputed metrics excluding admissions in the PCU, to avoid weighting from predictions for predominantly chronically ill patients with prolonged length of stay, who may not benefit from the same predictive situational awareness tools.

## Results

During the 90-day deployment, we observed 1369 PICU stays which resulted in 1132 PICU admissions (*n* = 1005 patients) after applying the age exclusion and merging consecutive stays. We observed 363 CDEs (*n* = 70 patients or admissions) with an admission-level CDE prevalence of 0.05. We excluded from our analysis 17 ineligible CDEs occurring within the first hour of the PICU admission. After applying 24-hour post-CDE censoring, 99 eligible CDEs experienced by 62 patients remained (Figure 1). Table 1 provides demographic characteristics among groups with and without eligible CDEs.

**Table 1.** Demographic characteristics of the PICU deployment cohort (January 7, 2026-April 7, 2026).

	Experienced no CDE (%)	Experienced at least 1 CDE (%)	Odds ratio (95% CI)
**Number of patients**	943	62	
**Sex = Female, *n* (%)**			
** Yes**	410 (43.5)	25 (40.3)	0.88 (0.52-1.47)
**No**	533 (56.5)	37 (59.7)	1 [Reference]
**Race, *n* (%)**			
**White**	401 (42.5)	29 (46.8)	1 [Reference]
**Black**	288 (30.5)	15 (24.2)	0.72 (0.37-1.35)
**Other**^a^	212 (22.5)	16 (25.8)	1.04 (0.54-1.94)
**Unknown**^b^	26 (2.8)	2 (3.2)	1.06 (0.17-3.81)
**Multiple**^c^	16 (1.7)	0 (0)	NA
**Hispanic or Latino ethnicity, *n* (%)**			
**Yes**	158 (16.8)	14 (22.6)	1.46 (0.76-2.66)
**No**	760 (80.6)	46 (74.2)	1 [Reference]
**Unknown**^b^	25 (2.7)	2 (3.2)	1.32 (0.21-4.63)
**Age category, *n* (%)** ^d^			
**Neonate (< 28 days)**	7 (0.7)	2 (3.2)	6.60 (0.94-29.60)
** Infant (>27 days, <2 years)**	260 (27.6)	24 (38.7)	2.13 (1.15-4.01)
**Child (2-11 years)**	439 (46.6)	19 (30.6)	1 [Reference]
**Adolescent (12-18 years)**	218 (23.1)	14 (22.6)	1.48 (0.72-3.00)
**Adult (19-24 years)**	19 (2.0)	3 (4.8)	3.65 (0.81-11.93)
**Insurance category, *n* (%)** ^d^			
**Private**	386 (40.9)	28 (45.2)	1 [Reference]
**Medicaid**	523 (55.5)	31 (50.0)	0.82 (0.48-1.39)
**Medicare**	1 (0.1)	0 (0)	NA
**Other**	16 (1.7)	3 (4.8)	2.58 (0.58-8.35)
**Unknown**	17 (1.8)	0 (0)	NA

Table presenting the demographic data of patients seen in the PICU during January 7, 2026 to April 7, 2026 P-WIN deployment period, categorized by whether the patient experienced a CDE. Patient age was computed based on when they were first seen in the PICU during the deployment period. None of the odds ratios presented in the rightmost column were statistically significant at a level of *P* < .05.

CDE, critical deterioration event.

A composite of either 1. CPR, 2. initiation of ECMO, 3. administration of bolus dose of “dilute” (10 μg/mL concentration) epinephrine, or 4. unplanned endotracheal intubation.

a “Other” race category contains patients whose self-reported race in the EHR was “Asian,” “Indian,” “Native Hawaiian or Other Pacific Islander,” “American Indian, Native American, or Alaskan Native,” “Middle Eastern (eg, Armenian, Iraqi, Lebanese)”, “Other,” or “Hispanic/Latino/Latinx.”

b “Unknown” Race and Ethnicity categories comprise patients whose self-reported race in the EHR was “Choose not to disclose,” “Unknown,” “Asked but unknown,” or “Refused.”

c “Multiple” category for Race consists of patients who had several different race or ethnicity records in the EHR.

d For patients with multiple PICU admissions, we used the age and payor category corresponding to the first PICU admission during the deployment period.

### Predictive model performance

P-WIN detected 73% of these 99 CDEs 1-24 hours in advance, significantly higher than PICU Warning Tool (39% sensitivity; *P* < .05) and huddle-based alerts (23% sensitivity; *P* < .05), and the earliest P-WIN alert triggered a median of 15.45 hours (IQR, 11.40-18.86) in advance. P-WIN had similar median timeliness to the PICU Warning Tool among the CDEs they both detected (*P* = .58), and apparently higher median timeliness than huddle alerts, but this difference was not significant (*P* = .12)—see Table S1. P-WIN’s admission-level PPV of 0.12 (95% CI, 0.10-0.14) was similar to PICU Warning Tool (0.12 [0.08-0.16]; *P* = .94) and huddle alerts (0.11 [0.06-0.17]; *P* = .82)—see Table 2. This corresponds to an NNA of 8.33 (95% CI, 6.98-10.34). During the 90-day period, 5% of admissions contained eligible CDEs and the median PICU admission length was 46.5 hours (IQR, 24.5-129.1 hours).

**Table 2.** Comparison of metrics for P-WIN and the PICU Warning Tool and huddle alert comparators.

	P-WIN model (550 variables) [Reference]	PICU Warning Tool	Huddle	Parsimonious P-WIN model (12 variables)
**CDE-level sensitivity (95% CI)**	**0.73** **(0.64-0.81)**	0.39 (0.30-0.48)^a^	0.23 (0.15-0.31)^a^	0.69 (0.60-0.78)
**Admission-level PPV (95% CI)**	**0.12** **(0.10-0.14)**	**0.12** **(0.08-0.16)**	0.11 (0.06-0.17)	0.10 (0.08-0.13)
**Alert-level PPV (95% CI)**	0.04 (0.03-0.05)	0.03 (0.02-0.03)^b^	**0.10** **(0.07-0.13)** ^b^	0.04 (0.03-0.05)
**Median timeliness, hours (IQR)**	15.45 (11.40-18.86)	**17.40** **(11.48-19.72)**	10.33 (5.74-15.02)	15.02 (9.00-18.40)
**Average of alerts per patient-day across admissions (95% CI)**				
**Overall**	0.21 (0.18-0.23)	**0.12** **(0.10-0.15)** ^c^	N/A	0.21 (0.18-0.24)
**Among admissions w/CDE**	1.00 (0.85-1.14)	**0.54** **(0.39-0.70)** ^c^	N/A	0.99 (0.85-1.12)
**Among admissions w/out CDE**	0.15 (0.13-0.18)	**0.09** **(0.07-0.12)** ^c^	N/A	0.16 (0.13-0.18)

CDE-level sensitivity = (Number of CDE which were detected) / (Number of CDE overall).

Admission-level PPV = (Number of admissions for which the first CDE was detected) / (Number of admissions with alerts).

Alert-level PPV = (Number of alerts detecting subsequent CDE) / (Number of alerts).

Median timeliness = The median of the lead time of the earliest alert within the 1-24 hour period prior to the CDE, among those CDEs with alerts in that period.

A CDE was detected if it had an alert within the preceding 1-24 hours.

a Indicates a statistically significant difference in sensitivity (*P* < .05) using McNemar’s test.

b Indicates a statistically significant difference in PPV (*P* < .05) using a permutation test (*N* = 10 000 iterates).

c Indicates a statistically significant difference in alerts per patient-day (*P* < .05) using Wilcoxon’s signed rank test.

CDE, critical deterioration event, which includes CPR, initiation of ECMO, administration of bolus dose of “dilute” (10 μg/mL concentration) epinephrine (Epinephrine), or unplanned endotracheal intubation (Intubation).

Bold values indicate the best performance across systems for the given metric.

The median number of hours between admission start and the CDE time was 150.68 (IQR, 42.55-371.78) among the CDEs that P-WIN detected 1-24 hours in advance was significantly larger than the median value of 24.87 (IQR, 7.87-128.86; *P* < .05) among CDEs not detected. P-WIN detected 80% of CDEs occurring at least 16 hours after arrival in the PICU.

P-WIN generated 0.21 (95% CI, 0.18-0.23) alerts per patient-day, compared to 0.12 (95% CI, 0.10-0.15) for PICU Warning Tool, *P* < .05. P-WIN had an alert-level PPV (0.04 [95% CI, 0.03-0.05]) that was higher than PICU Warning Tool (0.03 [95% CI, 0.02-0.03]; *P* < .05), and lower than huddle alerts (0.10 [95% CI, 0.07-0.13]; *P* < .05).

Among 62 PICU admissions having CDE(s), P-WIN alerted at least 1 hour in advance of the first CDE for 41 admissions and 1-24 hours in advance for 36 admissions. 24 admissions had their first P-WIN alert more than 24 hours in advance of the first CDE (Figure S2).

P-WIN was moderately well calibrated, achieving a Brier Skill Score of 0.14 (95% CI, 0.04-0.23). Calibration plots using 20 equal-frequency bins (Figure S3a) and 10 equal-width bins (Figure S3b) exhibit some overconfidence in higher predictive probabilities and underconfidence in lower predictive probabilities. Imperfect probability calibration is acceptable in P-WIN’s intended use case, namely triggering alerts at risk threshold(s) pre-selected to balance sensitivity and alert burden.

Counts of each CDE type detected by P-WIN and the comparators are summarized in Table 3, and the median timeliness for detected events within each type can be found in Table S2. P-WIN detected the majority of CDEs of each type which were also detected by PICU Warning Tool or huddle alerts, missing only 2 CPR events, 6 epinephrine events, and 4 intubation events which were detected by either comparator system (Figure S4). For these counts, 24-hour event censoring was only instituted between events of the same type.

**Table 3.** Counts of CDEs detected, by CDE type, for the P-WIN model, the 12-variable parsimonious P-WIN model, and the comparators.

		*N* (%) of CDE detected			
CDE type	Number observed	P-WIN model (550 variables)	PICU Warning Tool	Huddle	Parsimonious P-WIN model (12 variables)
**CPR**	10	**8 (80)**	4 (40)	5 (50)	7 (70)
**ECMO**	4	**4 (100)**	2 (50)	1 (25)	**4 (100)**
**Epinephrine**	78	**63 (81)**	44 (56)	25 (32)	61 (78)
**Intubation**	47	**33 (72)**	11 (23)	9 (19)	30 (64)

For each CDE type, CDEs were censored if they occurred < 24 hour after a prior CDE of the same type, and censored CDEs are not included in this table.

CDE = Critical Deterioration Event, which includes cardiopulmonary resuscitation (CPR), initiation of extracorporeal membrane oxygenation (ECMO), administration of bolus dose of “dilute” (10 μg/mL concentration) epinephrine (Epinephrine), or unplanned endotracheal intubation (Intubation).

Bold values indicate the highest sensitivity across systems for the given CDE type.

The risk threshold chosen to achieve 80% sensitivity in the 2021-2022 retrospective alert burden analysis^12^ provided significantly lower sensitivity (*P* < .05), significantly higher PPV (*P* < .05), significantly lower alerts per patient-day (*P* < .05), and similar median timeliness compared to the risk thresholds achieving 90% and 95%, respectively—see Table S3.

Using an alert threshold matching the alert burden of PICU Warning Tool, P-WIN still achieved higher sensitivity (*P* < .05). When chosen to match the sensitivity of PICU Warning Tool, P-WIN achieved lower average alert burden (*P* < .05)—see Table S4.

### Operational statistics during deployment

During the deployment period, PICU departments saw a median of 107 distinct patients per calendar day (IQR, 103-109), with a median census of 96 patients at any given time point (IQR, 94-97). The median duration of iterations of our pipeline (including API requests, feature extraction, model inference, and saving results to a SQL database) was 8.17 minutes (IQR, 4.77-8.90 minutes). Overall, 98% of iterations finished in under 15 minutes and only 18 iterations (0.4%) exceeded 30 minutes. The entire process was run on a single CPU core of an Intel Xeon Silver 4214 2.20 GHz processor. We did not measure memory usage during the prospective deployment period, but post hoc experiments suggest the usage does not exceed 500 MB. We did not have the ability to measure the backend load on the Epic interconnect server during this deployment. Model predictions were not streamed back to the EHR, and were archived on a research server for evaluation purposes.

### Model fairness

P-WIN exhibited fairness across demographic subgroups (Table S5). There were no statistically significant differences in CDE-level sensitivity between across the subgroups: males (0.72 [95% CI, 0.60-0.83]) vs females (0.73 [95% CI, 0.59-0.88], *P* = 1.0); white patients (0.83 [95% CI, 0.72-0.93]) vs black patients (0.57 [95% CI, 0.33-0.81], *P* = .07) or patients of other races (0.69 [95% CI, 0.50-0.85], *P* = .24); patients of non-Hispanic/Latino (0.72 [95% CI, 0.61-0.82]) vs Hispanic/Latino ethnicity (0.77 [95% CI, 0.59-0.95], *P* = .1.0); Private insurance (0.76 [95% CI, 0.63-0.87]) vs Medicaid (0.71 [95% CI, 0.58-0.83], *P* = .64). Subgroups represented by fewer than 10 CDEs were omitted from this analysis.

### Performance of the parsimonious P-WIN models

Among the 12-feature, 24-feature, and 42-feature parsimonious versions of the P-WIN model, none had statistically significantly different event-level sensitivity, admission-level PPV, alert-level PPV, or average alert burden from the full P-WIN model at a level of *P* < .05 (Table S6). For each CDE type, the full P-WIN model detected at least as many CDEs 1-24 hours in advance as all of the parsimonious models (Table S7), and all parsimonious models outperformed the PICU Warning Tool and huddle assessments in every CDE category.

### Performance when excluding admissions in the PCU

Out of 1132 admissions involving 1005 patients, 1071 PICU admissions involving 964 patients occurred outside of the PCU. Limiting to these non-PCU admissions resulted in similar sensitivity to the main analysis, somewhat higher PPV for all alert workflows and slightly lower average alert burden for both P-WIN and the PICU Warning Tool (Table S8).

### Risk trajectory for a sample patient who experienced CDE

A sample patient trajectory leading up to several CDEs is shown in Figure 2A, with P-WIN alerting 62 minutes in advance. Figure 2B shows the top 10 driving factors contributing to the alert, ranked in descending order of SHAP values, and Figure S5 shows an analogous plot for the 12-variable parsimonious version of P-WIN. The high-dimensional model attributed risk to several clinically meaningful variables that are ranked high but absent from the parsimonious model (as well as the 24-variable and 42-variable parsimonious models), including pulse, respiratory rate, cuff mean arterial pressure, and oxygen saturation (SpO_2_). An overall plot of P-WIN model variables, ranked by mean of the absolute SHAP values across a large cohort, can be found in the manuscript describing P-WIN’s development.^12^

**Figure 2.**
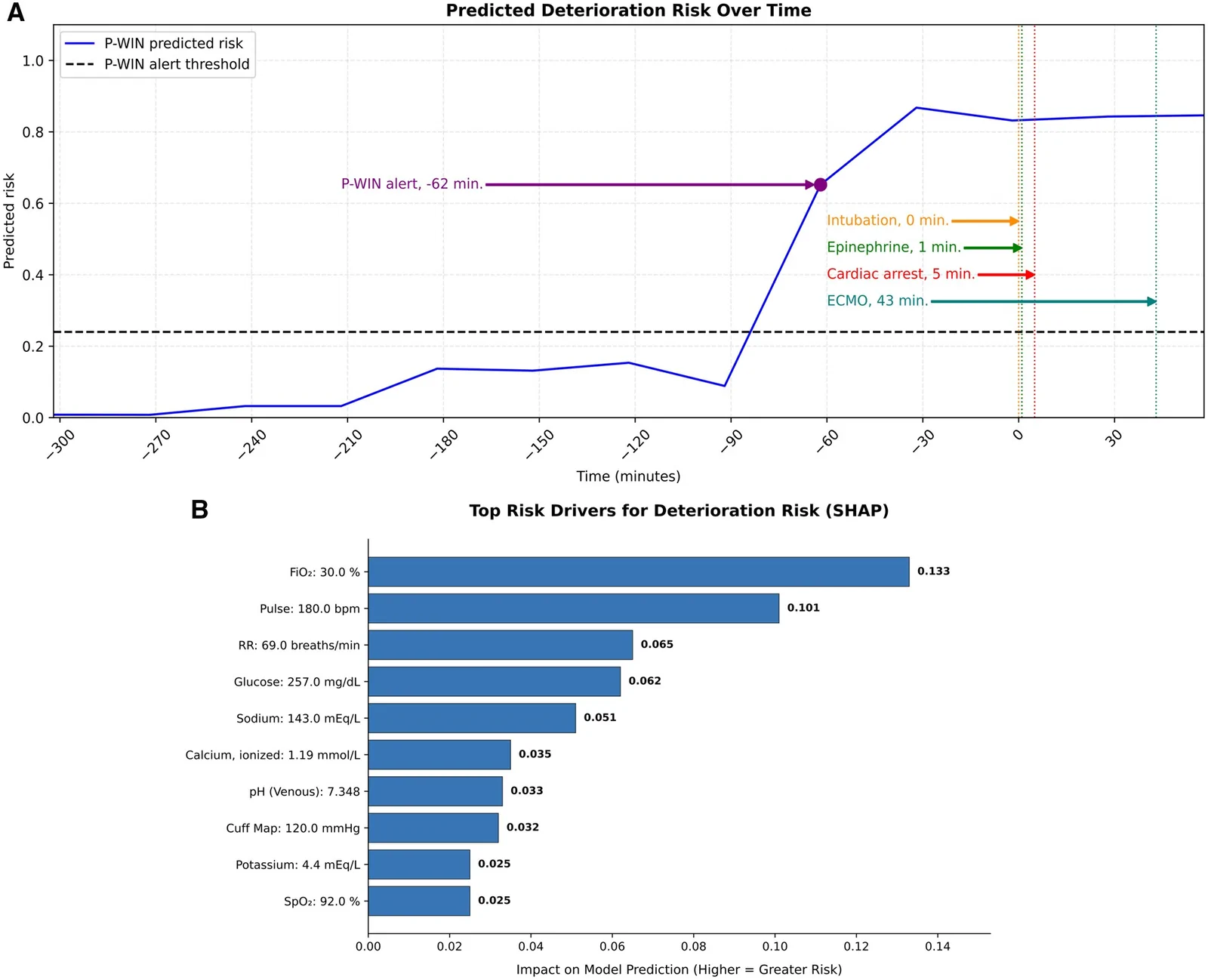
The P-WIN predicted risk levels in the hours leading up to a patient experiencing several CDEs, and the top 10 risk factors driving a P-WIN alert. P-WIN alerted 62 minutes prior to the unplanned intubation, as shown in part A. Part B shows the top 10 variables contributing to the elevated risk level at the time of the P-WIN alert, ranked in descending order of absolute marginal contribution to the predicted risk for this patient. The values expressed were computed based on SHAP values but mapped to the probability space. The subsequent CDEs were censored from the main evaluation, as they occurred in quick succession after the initial event. A second epinephrine bolus at 2 minutes was omitted from the plot (a), for clarity. Time is measured in minutes, where *t* = 0 corresponds to the first CDE, an unplanned endotracheal intubation.

## Discussion

In a real-time silent prospective deployment in a large quaternary-care PICU, P-WIN improved early detection of CDEs in a live clinical environment. P-WIN detected almost twice as many CDEs as PICU Warning Tool and over 3 times as many as huddle alerts, including 8 out of 10 observed cardiac arrests, while maintaining similar admission-level PPV with these comparators (all 0.11-0.12). Although the pre-selected P-WIN alert threshold generated a higher average alert burden than the PICU Warning Tool, P-WIN maintained significantly higher sensitivity (61% vs 39%; *P* < .05) using a threshold matching the alert burden of the PICU Warning Tool. These findings demonstrate that in a real‑world clinical environment, P‑WIN can provide markedly improved sensitivity without increasing alert fatigue.

P-WIN should also be considered within the broader landscape of deterioration prediction systems. Other EHR-based systems have demonstrated that deterioration can be identified hours in advance.^7–10^ A key contribution of this study is the assessment of P-WIN’s real-time operational characteristics including alerts per patient-day and alert-level PPV, metrics not consistently addressed in prior model evaluations.

An alert-level PPV of 0.04 may be inappropriate for a fully interruptive alerting system. However, implementation decisions should be guided by alert burden over time rather than by PPV alone. P-WIN generated 0.21 alerts per patient-day at the primary risk threshold, and the 12-hour refractory period was included to mitigated repeated alerts during sustained high-risk periods; the burden can be further adjusted through threshold selection. Because P-WIN provides a continuous risk score, it is also well-suited to a tiered implementation strategy in which lower-risk predictions support situational awareness through non-interruptive displays or huddles, while only the highest-risk patients trigger active notifications. We are currently evaluating alert burden and workflow integration within our PICU to identify thresholds that are clinically effective and operationally sustainable risk tiers.

High-frequency monitor data have been used to predict deterioration in adult, neonatal, and specialized pediatric critical-care populations.^24–26^ P-WIN was intentionally designed for deployment through standard Epic interoperability interfaces, but future work will evaluate whether augmenting P-WIN with high-frequency bedside monitor signals can further improve predictive performance, timeliness, and alert precision.

P-WIN’s median timeliness should be interpreted alongside its alert frequency and alert-level PPV. As seen in Figure S2, some PICU admissions had P-WIN alerts essentially every 12 hours in the 72-hour period prior to the first CDE (the highest frequency allowed by the refractory policy), suggesting a persistent elevated risk state–and many had an alert very early in the 24 hours prior to the CDE.

Another important contribution of this study is demonstrating technical feasibility for real-time ML deployment at scale. P-WIN integrates 550 EHR-derived personalized variables and generates predictions every 30 minutes using a combination of FHIR and proprietary Epic APIs. This deployment process ran on a single CPU core with modest memory needs, and pull and process all data in 8-9 minutes. As this system is converted to a production-level tool, attention to robustness of the data retrieval pipeline will be crucial.

We were unable to rely entirely on FHIR APIs, as the Epic on FHIR catalog^27^ does not include API endpoints for retrieving a real-time patient census for a department list or a record of medication administration events. This presents a development opportunity, as such functionality is necessary for such a real-time deployment.

We also demonstrated that parsimonious versions of P-WIN approximated the real-time prospective performance of the full P-WIN model. Although P-WIN detected at least as many CDEs in each category, the parsimonious models could be practical and viable replacements in settings with limited technical infrastructure or computational resources.

Beyond displaying the strongest predictive performance, the high-dimensional P-WIN model also provides the opportunity to identify highly personalized key risk factors via SHAP analysis which may support hypothesis generation and discussions about clinical interventions. Several clinically meaningful top risk factors for the sample patient demonstrated in Figure 2 are absent from the parsimonious model, suggesting that simpler models may provide less individualized explanations. Future studies should address the clinical utility of personalized risk explanations.

The P-WIN model did not demonstrate performance bias with respect to sex, race, or ethnicity based on the 2021-2022 external validation cohort^12^, and we did not detect statistically significant demographic bias in CDE-level sensitivity during this 90-day deployment. The relatively small number of CDEs in certain groups limits definitive conclusions, and ongoing monitoring for drift and fairness concerns will be essential to fully assess P-WIN’s long-term performance and fairness characteristics. Future work will assess how sustained use of P-WIN affects decision-making, workflow efficiency, shared situational awareness, and ultimately patient outcomes. Integrating P‑WIN into existing practices and the EHR system will require attention to communication strategies, clinician education, and user interface design to ensure that predictions are interpretable and actionable.

### Limitations

This prospective study has several limitations. First, generalizability is not addressed, as the model was developed and deployed at the same site. This study explores both high-dimensional and parsimonious models which could be evaluated across institutions in future work. Second, a longer deployment period may reveal additional seasonality effects and would enable more robust estimates of performance and operational characteristics. Third, although P-WIN demonstrated significantly higher alert-level PPV than the PICU Warning Tool, the absolute PPV remained low (4%), corresponding to an NNA of approximately 25. This reflects the intrinsic difficulty of predicting rare but clinically important CDEs. Fourth, as P-WIN was deployed “silently,” the present study does not address the complexities of workflow integration and clinician feedback. Fifth, the statistical power of demographic fairness tests was limited by the small number of CDE in some demographic groups, warranting additional fairness testing during a longer deployment period. Sixth, the success of model deployment hinges on the operational robustness of data-retrieval APIs and capacity of the backend Epic interconnect server. We encountered intermittent failures of API requests during the 90-day deployment, and these issues should be addressed prior to clinician-facing production-level deployment. Additionally, we did not measure load on the backend interconnect server during this deployment. Seventh, P-WIN was developed using data from PICU stays in 2014-2020, which may affect its ability to generalize to new data due to temporal shifts. We are currently working to update the P-WIN model using data from more recent PICU stays. Lastly, the extent to which the high-dimensional P-WIN model provided more clinically meaningful or actionable feature explanations was not systematically evaluated in this study. Future work may consider differences in interpretability and clinical utility across models of varying complexity, to determine whether such differences translate into improved clinical decision support or model trust.

## Conclusions

In this real-time prospective deployment evaluation, P-WIN outperformed 2 existing deterioration warning workflows. Its ability to anticipate deterioration in the PICU underscores its potential to enhance situational awareness. These findings illustrate the feasibility and clinical value of integrating personalized ML-based risk prediction into critical care decision support, and opens the door to real-time clinical deployment of high-dimensional models.

## Supplementary Material

ooag202_Supplementary_Data

## Data Availability

The data used in this study are owned by Children’s Hospital of Philadelphia (CHOP) and contain sensitive patient information. Due to institutional and privacy regulations, these data cannot be shared publicly. Researchers interested in accessing the data must obtain approval from CHOP and comply with all applicable data use agreements and ethical guidelines. The model’s data dictionary can be shared upon request. The underlying code for this study is not publicly available but may be made available to qualified researchers on reasonable request from the corresponding author, pending CHOP approval.
