# Supplementary material for: P-WIN deployment: real-time personalized machine learning to predict critical deterioration in a pediatric intensive care unit
Source: JAMIA Open. 2026 Oct 19;9(5):ooag202. doi: 10.1093/jamiaopen/ooag202 (PMC13680097; doi:10.1093/jamiaopen/ooag202)
Supplement: Supplementary file 1 [file ooag202_supplementary_data.docx]

Supplemental Digital Content

[eMethods	1](#_Toc240446610)

[API Data Retrieval Sources	1](#_Toc240446611)

[Data Preprocessing and Feature Extraction	2](#_Toc240446612)

[eTABLES	2](#_Toc240446613)

[**eTable 1**. Direct comparison of median alert timeliness in hours between P-WIN and the PICU Warning Tool and Huddle systems.	2](#_Toc240446614)

[**eTable 2**. Median alert timeliness, by CDE type, for P-WIN and the comparators.	2](#_Toc240446615)

[**eTable 3.** Sensitivity analysis of P-WIN prospective performance using three alert thresholds.	3](#_Toc240446616)

[**eTable 4**. Comparison of two alternative P-WIN thresholds to the PICU Warning Tool.	3](#_Toc240446617)

[**eTable 5.** CDE-level sensitivity performance of the P-WIN model within demographic groups.	4](#_Toc240446618)

[**eTable 6.** Comparison of metrics for the full P-WIN model and the parsimonious versions.	5](#_Toc240446619)

[**eTable 7.** Counts of CDEs detected, by CDE type, for P-WIN, PICU Warning Tool, Huddle alerts, and the parsimonious models.	5](#_Toc240446620)

[**eTable 8.** Comparison of metrics for P-WIN and the PICU Warning Tool and huddle alert comparators, excluding admissions in the Progressive Care Unit (PCU).	6](#_Toc240446621)

[eFIGURES	7](#_Toc240446622)

[**eFigure 1.** Real-time data retrieval and processing workflow for the P-WIN model.	7](#_Toc240446623)

[**eFigure 2.** P-WIN alert profiles over time for the 41 PICU admissions which had P-WIN alerts at least 1 hour prior to their first CDE.	8](#_Toc240446624)

[**eFigure 3.** Calibration plots for the P-WIN model on the prospective deployment data using (a) 20 equal-frequency bins and (b) 10 equal-width bins. The labels on the equal-width bins indicate the number of predictions in each bin.	8](#_Toc240446625)

## eMethods

### API Data Retrieval Sources

FHIR APIs included those used to pull laboratory and flowsheet results (Observation.Search), medication orders (MedicationRequest.Search), and medication concept information (Medication.Read). Proprietary EPIC APIs included those used to pull department census information (GetUnitCensus2), convert patient medical record numbers (MRN) to encoded patient FHIR IDs (GetPatientIdentifiers), and pull medication administration (MAR) records (GetMedicationAdministrationHistory). The data retrieval process is illustrated in **eFigure 1.**

### Data Preprocessing and Feature Extraction

P-WIN Model variables were extracted from medication, flowsheet, and demographic features using the same methodology used during model development.^1^ Medication variables were based on RxNorm^2^ active ingredients and Veterans Administration (VA) Drug Classes^3^, lab variables were based on Logical Observation Identifiers Names and Codes (LOINC)^4^, and flowsheet variables were based on the measurement’s flowsheet name. Each medication variable (VA class or active ingredient) consisted of a binary flag indicating whether or not the corresponding medication was administered in the 4-hour window prior to the prediction time. For each lab result or numerical flowsheet element, we derived a numerical feature consisting of the median value over the 30-minute window prior to the prediction horizon, where null values were forward-filled for 4 hours. Variables for categorical flowsheet elements consisted of the most recent value, with null values again forward-filled for 4 hours.

## eTABLES

**eTable 1**. Direct comparison of median alert timeliness in hours between P-WIN and the PICU Warning Tool and Huddle systems.

| **Comparison** | **N of CDE detected by both systems** | **P-WIN median timeliness (IQR), hours** | **Comparator median timeliness (IQR), hours** |
| --- | --- | --- | --- |
| P-WIN vs. PICU Warning Tool | 35 | **17.53 (12.68-19.07)** | 17.40 (11.83-20.40) |
| P-WIN vs. Huddle | 18 | **15.62 (14.32-19.05)** | 12.00 (9.63-16.07) |

Median timeliness = The median of the lead time of the earliest alert within the 1-24hr period prior to the CDE, among those CDEs with alerts in that period.

For each comparison, the medians and interquartile ranges (IQR) were measured within the sets of CDEs detected by both models 1-24 hours in advance. CDEs occurring within 24 hours after a prior CDE were excluded (censored).

P-WIN’s timeliness was not statistically significantly different than either PICU Warning Tool (P = 0.58) or Huddle alerts (P = 0.12) according to Wilcoxon’s signed rank test.

**eTable 2**. Median alert timeliness, by CDE type, for P-WIN and the comparators.

|  | **Median timeliness, hours (IQR)** | | | | | |
| --- | --- | --- | --- | --- | --- | --- |
| **CDE type** | **P-WIN** | **PICU**  **Warning Tool** | **Huddle** | **Parsimonious P-WIN models** | | |
|  |  |  |  | **42-variable** | **24-variable** | **12-variable** |
| CPR | 12.74  (4.31-15.78) | 14.14  (10.79-15.48) | 9.07  (1.56-9.63) | **14.60**  **(8.29-15.25)** | 14.49  (4.31-15.21) | 11.68  (4.29-14.96) |
| ECMO | **11.25**  **(2.69-20.08)** | 6.63  (3.98-9.27) | 10.25  (10.25-10.25) | 10.25  (2.69-18.58) | 10.25  (2.69-18.58) | 8.25  (2.69-15.58) |
| Epinephrine | 14.57  (9.75-18.82) | 13.95  (9.32-18.89) | 13.37  (3.88-16.32) | 15.17  (10.24-18.54) | 15.13  (9.61-18.42) | **15.18**  **(8.48-18.37)** |
| Intubation | 15.95  (6.87-18.85) | **17.40**  **(11.69-20.45)** | 9.88  (7.6-12.5) | 14.96  (7.41-18.92) | 13.17  (8.15-18.57) | 13.36  (6.68-17.80) |

For each CDE type, CDE were censored if they occurred < 24hr after a prior CDE of the same type, and censored CDE are not included in this table.

CDE = Critical Deterioration Event, which includes cardiopulmonary resuscitation (CPR), initiation of extracorporeal membrane oxygenation (ECMO), administration of bolus dose of “dilute” (10 mcg/ml concentration) epinephrine (Epinephrine), or unplanned endotracheal intubation (Intubation).

**eTable 3.** Sensitivity analysis of P-WIN prospective performance using three alert thresholds.

|  | **P-WIN Threshold A**  **[Reference]** | **P-WIN Threshold B** | **P-WIN Threshold C** |
| --- | --- | --- | --- |
| **CDE-level sensitivity (95% CI)** | 0.73 (0.64-0.81) | 0.86 (0.78-0.93)^a^ | **0.90 (0.83-0.96)** **^a^** |
| **Admission-level PPV (95% CI)** | **0.12 (0.10-0.14)** | 0.09 (0.08-0.11)^b^ | 0.07 (0.06-0.07) ^b^ |
| **Alert-level PPV (95% CI)** | **0.04 (0.03-0.05)** | 0.03 (0.02-0.03) ^b^ | 0.02 (0.02-0.02) ^b^ |
| **Median timeliness, hours (IQR)** | 15.45 (11.40-18.86) | **15.17 (11.43-19.48)** | 15.42 (11.43-19.70) |
| **Average of alerts per patient-day across admissions (95% CI)** |  |  |  |
| **Overall** | **0.21 (0.18-0.23)** | 0.42 (0.37-0.47)^c^ | 0.78 (0.72-0.83) ^c^ |
| **Among admissions w/ CDE** | **1.00 (0.85-1.14)** | 1.28 (1.13-1.42) ^c^ | 1.57 (1.45-1.70) ^c^ |
| **Among admissions w/out CDE** | **0.15 (0.13-0.18)** | 0.36 (0.31-0.41) ^c^ | 0.72 (0.67-0.79) ^c^ |

Thresholds A, B, and C were chosen based on having detected 80%, 90%, and 95% of CDE, respectively, 1-24 hours in advance during the 2021-2022 retrospective analysis. Threshold A is the default P-WIN risk threshold used in the main results.

Event-level sensitivity = (# CDE which were detected) / (# CDE overall)

Admission-level PPV = (# of admissions for which the first CDE was detected) /(# admissions with alerts)

Alert-level PPV = (# of alerts detecting subsequent CDE)/ (# of alerts)

Median timeliness = The median of the lead time of the earliest alert within the 1-24hr period prior to the CDE, among those CDEs with alerts in that period.

A CDE was detected if it had an alert within the preceding 1-24 hours.

^a^Indicates a statistically significant difference in sensitivity (P < 0.05) using McNemar’s test.

^b^Indicates a statistically significant difference in PPV (P < 0.05) using a permutation test (N = 10,000 iterates).

^c^Indicates a statistically significant difference in alerts per patient-day (P < 0.05) using Wilcoxon’s signed rank test.

CDE = Critical Deterioration Event, which includes cardiopulmonary resuscitation (CPR), initiation of extracorporeal membrane oxygenation (ECMO), administration of bolus dose of “dilute” (10 mcg/ml concentration) epinephrine (Epinephrine), or unplanned endotracheal intubation (Intubation).

**eTable 4**. Comparison of two alternative P-WIN thresholds to the PICU Warning Tool.

|  | **P-WIN Threshold D** | **P-WIN Threshold E** | **PICU Warning Tool**  **[Reference]** |
| --- | --- | --- | --- |
| **CDE-level sensitivity (95% CI)** | 0.39 (0.30-0.49) | 0.61 (0.0.52-0.71)^a^ | 0.39 (0.30-0.48) |
| **Admission-level PPV (95% CI)** | 0.13 (0.08-0.19) | **0.13 (0.09-0.17)** | 0.12 (0.08-0.16) |
| **Alert-level PPV (95% CI)** | **0.08 (0.06-0.10)** | 0.05 (0.04-0.06) | 0.03 (0.02-0.03) |
| **Median timeliness, hours (IQR)** | 16.00 (9.52-18.83) | 14.40 (11.51-18.40) | **17.40 (11.48-19.72)** |
| **Average of alerts per patient-day across admissions (95% CI)** |  |  |  |
| **Overall** | **0.05 (0.03-0.06)^c^** | 0.12 (0.10-0.15) | 0.12 (0.10-0.15) |
| **Among admissions w/ CDE** | **0.42 (0.30-0.54)** | 0.76 (0.62-0.91) ^c^ | 0.54 (0.39-0.70) |
| **Among admissions w/out CDE** | **0.02 (0.01-0.03) ^c^** | 0.08 (0.06-0.10) | 0.09 (0.07-0.12) |

P-WIN thresholds D and E were chosen to achieve similar CDE-level sensitivity and average alert burden, respectively, to the PICU Warning Tool during this prospective deployment.

Event-level sensitivity = (# CDE which were detected) / (# CDE overall)

Admission-level PPV = (# of admissions for which the first CDE was detected) /(# admissions with alerts)

Alert-level PPV = (# of alerts detecting subsequent CDE)/ (# of alerts)

Median timeliness = The median of the lead time of the earliest alert within the 1-24hr period prior to the CDE, among those CDEs with alerts in that period.

A CDE was detected if it had an alert within the preceding 1-24 hours.

^a^Indicates a statistically significant difference in sensitivity (P < 0.05) using McNemar’s test.

^b^Indicates a statistically significant difference in PPV (P < 0.05) using a permutation test (N = 10,000 iterates).

CDE = Critical Deterioration Event, which includes cardiopulmonary resuscitation (CPR), initiation of extracorporeal membrane oxygenation (ECMO), administration of bolus dose of “dilute” (10 mcg/ml concentration) epinephrine (Epinephrine), or unplanned endotracheal intubation (Intubation).

**eTable 5.** CDE-level sensitivity performance of the P-WIN model within demographic groups.

|  | **N (%) of CDE** | **Sensitivity**  **(95% CI)** | **P-value**  **(Fisher’s exact test)** |
| --- | --- | --- | --- |
| **Sex** |  |  |  |
| Female | 41 (41) | 0.73 (0.59-0.88) | 1.0 |
| Male | 58 (59) | 0.72 (0.60-0.83) | Reference |
| **Race** |  |  |  |
| Black | 21 (21) | 0.57 (0.33-0.81) | 0.07 |
| White | 46 (46) | 0.83 (0.72-0.93) | Reference |
| Other ^a^ | 26 (26) | 0.69 (0.50-0.85) | 0.24 |
| **Hispanic or Latino Ethnicity** |  |  |  |
| Yes | 22 (22) | 0.77 (0.59-0.95) | 1.0 |
| No | 71 (71) | 0.72 (0.61-0.82) | Reference |
| **Insurance Category** |  |  |  |
| Private | 46 (45) | 0.76 (0.63-0.87) | Reference |
| Medicaid | 48 (46) | 0.71 (0.58-0.83) | 0.64 |

The CDE-level sensitivity of the P-WIN model within demographic groups during the January 7 – April 7, 2026 deployment period is shown. Only groups containing at least 10 CDE are included.

CDE = Critical Deterioration Event, which includes cardiopulmonary resuscitation (CPR), initiation of extracorporeal membrane oxygenation (ECMO), administration of bolus dose of “dilute” (10 mcg/ml concentration) epinephrine (Epinephrine), or unplanned endotracheal intubation (Intubation).

^a^ The “Other” race category contains patients whose self-reported race in the EHR was “Asian”, “Indian”, “Native Hawaiian or Other Pacific Islander”, “American Indian, Native American, or Alaskan Native”, “Middle Eastern (e.g., Armenian, Iraqi, Lebanese)”, “Other”, or “Hispanic/Latino/Latinx”.

**eTable 6.** Comparison of metrics for the full P-WIN model and the parsimonious versions.

|  | **P-WIN**  **[Reference]** | **Parsimonious models** | | |
| --- | --- | --- | --- | --- |
|  |  | **42-variable** | **24- variable** | **12- variable** |
| **CDE-level sensitivity (95% CI)** | 0.73 (0.64-0.81) | 0.70 (0.61-0.79) | 0.70 (0.60-0.79) | 0.69 (0.60-0.78) |
| **Admission-level PPV (95% CI)** | 0.12 (0.09-0.14) | 0.10 (0.08-0.13) | 0.11 (0.08-0.13) | 0.10 (0.08-0.13) |
| **Alert-level PPV (95% CI)** | 0.04 (0.03-0.05) | 0.04 (0.03-0.05) | 0.04 (0.03-0.04) | 0.04 (0.03-0.05) |
| **Median timeliness, hours (IQR)** | 15.45  (11.40-18.86) | 16.12  (11.32-18.95) | 15.18  (10.93-18.72) | 15.02  (9.00-18.40) |
| **Average of alerts per patient-day across admissions (95% CI)** |  |  |  |  |
| **Overall** | 0.21 (0.18-0.23) | 0.21 (0.19-0.24) | 0.21 (0.18-0.24) | 0.21 (0.18-0.24) |
| **Among admissions w/ CDE** | 1.00 (0.85-1.14) | 0.99 (0.86-1.13) | 1.00 (0.87-1.14) | 0.99 (0.85-1.12) |
| **Among admissions w/out CDE** | 0.15 (0.13-0.18) | 0.16 (0.13-0.19) | 0.16 (0.13-0.18) | 0.16 (0.13-0.18) |

The 42-, 24-, and 12-feature parsimonious ensemble models were developed using top-k feature selectors for each horizon model, with k = 10, 20, and 30, respectively. Features were ranked according to mutual information with the CDE outcome.

CDE-level sensitivity = (# CDE which were detected) / (# CDE overall)

Admission-level PPV = (# of admissions for which the first CDE was detected)/(# admissions with alerts)

Alert-level PPV = (# of alerts detecting subsequent CDE)/(# of alerts)

Median timeliness = The median of the lead time of the earliest alert within the 1-24hr period prior to the CDE, among those CDEs with alerts in that period.

A CDE was detected if it had an alert within the preceding 1-24 hours.

^a^Indicates a statistically significant difference in sensitivity (P < 0.05) using McNemar’s test.

^b^Indicates a statistically significant difference in PPV (P < 0.05) using a permutation test (N = 10,000 iterates).

^c^Indicates a statistically significant difference in alerts per patient-day (P < 0.05) using Wilcoxon’s signed rank test.

CDE = Critical Deterioration Event, which includes cardiopulmonary resuscitation (CPR), initiation of extracorporeal membrane oxygenation (ECMO), administration of bolus dose of “dilute” (10 mcg/ml concentration) epinephrine (Epinephrine), or unplanned endotracheal intubation (Intubation).

**eTable 7.** Counts of CDEs detected, by CDE type, for P-WIN, PICU Warning Tool, Huddle alerts, and the parsimonious models.

|  |  | **N (%) of CDE detected** | | | | | |
| --- | --- | --- | --- | --- | --- | --- | --- |
| **CDE type** | **Number observed** | **P-WIN** | **PICU**  **Warning Tool** | **Huddle** | **Parsimonious P-WIN models** | | |
|  |  |  |  |  | **42-variable** | **24-variable** | **12-variable** |
| CPR | 10 | **8 (80)** | 4 (40) | 5 (50) | 7 (70) | **8 (80)** | 7 (70) |
| ECMO | 4 | **4 (100)** | 2 (50) | 1 (25) | **4 (100)** | **4 (100)** | **4 (100)** |
| Epinephrine | 78 | **63 (81)** | 44 (56) | 25 (32) | **63 (81)** | 62 (79) | 61 (78) |
| Intubation | 47 | **33 (72)** | 11 (23) | 9 (19) | 30 (64) | 29 (62) | 30 (64) |

For each CDE type, CDE were censored if they occurred < 24hr after a prior CDE of the same type, and censored CDE are not included in this table.

The 42-, 24-, and 12-feature parsimonious ensemble models were developed using top-k feature selectors for each horizon model, with k = 10, 20, and 30, respectively. Features were ranked according to mutual information with the CDE outcome.

CDE = Critical Deterioration Event, which includes cardiopulmonary resuscitation (CPR), initiation of extracorporeal membrane oxygenation (ECMO), administration of bolus dose of “dilute” (10 mcg/ml concentration) epinephrine (Epinephrine), or unplanned endotracheal intubation (Intubation).

**eTable 8.** Comparison of metrics for P-WIN and the PICU Warning Tool and huddle alert comparators, excluding admissions in the Progressive Care Unit (PCU).

|  | **P-WIN [Reference]** | **PICU Warning Tool** | **Huddle** |
| --- | --- | --- | --- |
| **CDE-level sensitivity (95% CI)** | **0.73 (0.64-0.81)** | 0.39 (0.30-0.48)^a^ | 0.24 (0.15-0.32)^a^ |
| **Admission-level PPV (95% CI)** | 0.13 (0.10-0.15) | 0.14 (0.09-0.19) | 0.12 (0.06-0.18) |
| **Alert-level PPV (95% CI)** | 0.05 (0.04-0.06) | 0.04 (0.03-0.05) | **0.11 (0.07-0.14)^b^** |
| **Median timeliness, hours (IQR)** | 15.47 (11.37-18.87) | **17.19 (11.43-19.13)** | 10.33 (5.74-15.02) |
| **Average of alerts per patient-day across admissions (95% CI)** |  |  |  |
| **Overall** | 0.21 (0.18-0.23) | **0.11 (0.09-0.14)^c^** | N/A |
| **Among admissions w/ CDE** | 1.01 (0.86-1.16) | **0.55 (0.40-0.71) ^c^** | N/A |
| **Among admissions w/out CDE** | 0.15 (0.12-0.18) | **0.09 (0.06-0.11) ^c^** | N/A |

CDE-level sensitivity = (# CDE which were detected) / (# CDE overall)

Admission-level PPV = (# of admissions for which the first CDE was detected) /(# admissions with alerts)

Alert-level PPV = (# of alerts detecting subsequent CDE)/ (# of alerts)

Median timeliness = The median of the lead time of the earliest alert within the 1-24hr period prior to the CDE, among those CDEs with alerts in that period.

A CDE was detected if it had an alert within the preceding 1-24 hours.

^a^Indicates a statistically significant difference in sensitivity (P < 0.05) using McNemar’s test.

^b^Indicates a statistically significant difference in PPV (P < 0.05) using a permutation test (N = 10,000 iterates).

^c^Indicates a statistically significant difference in alerts per patient-day (P < 0.05) using Wilcoxon’s signed rank test.

CDE = Critical Deterioration Event, which includes cardiopulmonary resuscitation (CPR), initiation of extracorporeal membrane oxygenation (ECMO), administration of bolus dose of “dilute” (10 mcg/ml concentration) epinephrine (Epinephrine), or unplanned endotracheal intubation (Intubation).

## eFIGURES

**eFigure 1.** Real-time data retrieval and processing workflow for the P-WIN model.

The flowchart depicts the automated pipeline for prospective deployment within the pediatric intensive care unit (PICU). Data were retrieved at 30-minute intervals from February 7 to April 7, 2026. The process included: (1) identifying the active patient cohort via a census API; (2) extracting clinical data using a combination of Fast Healthcare Interoperability Resources (FHIR) and proprietary Epic APIs; and (3) processing these data into derived variables for model inference.

FHIR APIs: Observation.Search, MedicationRequest.Search, Medication.Read.

Proprietary EPIC APIs: GetUnitCensus2, GetPatientIdentifiers, GetMedicationAdministrationHistory.

**eFigure 2.** P-WIN alert profiles over time for the 41 PICU admissions which had P-WIN alerts at least 1 hour prior to their first CDE.

Time is measured in hours, where t = 0 corresponds to the time at which the first CDE of the admission occurred. Red dots indicate the earliest P-WIN alert for each PICU admission, and black dots indicate subsequent P-WIN alerts. We have limited the timespan to 72 hours prior to the first CDE for readability, and red arrows indicate that the earliest P-WIN alert occurred more than 72 hours prior.

CDE = Critical Deterioration Event, which includes cardiopulmonary resuscitation (CPR), initiation of extracorporeal membrane oxygenation (ECMO), administration of bolus dose of “dilute” (10 mcg/ml concentration) epinephrine (Epinephrine), or unplanned endotracheal intubation (Intubation).

**eFigure 3.** Calibration plots for the P-WIN model on the prospective deployment data using (a) 20 equal-frequency bins and (b) 10 equal-width bins. The labels on the equal-width bins indicate the number of predictions in each bin.

One prediction for each of the 1132 PICU admission was selected: for admissions with CDEs, we selected the prediction time closest to 2 hours prior to the first CDE; for admissions without CDEs, we selected a random prediction time.

**eFigure 4.** Venn diagrams of counts of Critical Deterioration Events (CDE) of each type which were detected 1-24 hours in advance by P-WIN, the PICU Warning Tool (PWT), and Huddle alerts.

The types include (a) cardiopulmonary resuscitation (CPR), (b) initiation of extracorporeal membrane oxygenation (ECMO), (c) administration of bolus dose of “dilute” (10 mcg/ml concentration) epinephrine (Epinephrine), and (d) unplanned endotracheal intubation (Intubation).

**eFigure 5.** Top 10 driving factors for the parsimonious 12-variable P-WIN model for a sample patient.

The top 10 variables contributing to the elevated risk level from the parsimonious 12- variable version of the P-WIN model, for the same patient and at the same time point illustrated in **Figure 2**. Variables are ranked in descending order of absolute marginal contribution to the predicted risk for this patient. The values expressed were computed based on SHAP values, but mapped to the probability space.

Some EHR variables were not available for this patient at the time of prediction, indicated in the figure by “N/A”. During inference, these were imputed with the median values seen in the development cohort.

**REFERENCES**

1.	Sanjiv Mehta, E.T., Victor M Ruiz, Helen Lingyun Shi, Ryan W Morgan, Robert M Sutton, Akira Nishisaki, Fuchiang (Rich) Tsui, *Machine learning-based prediction of critical deterioation in the pediatric intensive care unit.* Critical Care Explorations, 2026 (in press).

2.	Nelson, S.J., et al., *Normalized names for clinical drugs: RxNorm at 6 years.* J Am Med Inform Assoc, 2011. **18**(4): p. 441-8.

3.	Carter, J.S., et al., *Categorical information in pharmaceutical terminologies.* AMIA Annu Symp Proc, 2006. **2006**: p. 116-20.

4.	McDonald, C.J., et al., *LOINC, a universal standard for identifying laboratory observations: a 5-year update.* Clin Chem, 2003. **49**(4): p. 624-33.
